# Supporting people with Motor Neuron Disease (MND) to make decisions about gastrostomy feeding tube placement: a survey of UK healthcare professionals’ practice and beliefs

**DOI:** 10.1080/21678421.2024.2314061

**Published:** 2024-02-09

**Authors:** Sean White, Alicia O’Cathain, Vanessa Halliday, Michael Bradburn, Christopher J. McDermott

**Affiliations:** 1 Division of Neuroscience, The University of Sheffield, Sheffield, UK; 2 Sheffield Centre for Health and Related Research (SCHARR), The University of Sheffield, Sheffield, UK, and; 3 School of Medicine and Population Health, The University of Sheffield, Sheffield, UK

**Keywords:** Motor neuron disease, amyotrophic lateral sclerosis, gastrostomy, decision-making, survey, multidisciplinary team

## Abstract

**Objective:**

Understand the practice and beliefs of healthcare professionals (HCPs) supporting the decision-making of people with MND (pwMND) about gastrostomy placement, including identifying differences between professions.

**Methods:**

An online cross-sectional survey disseminated to HCPs who support the decision-making of pwMND about gastrostomy placement.

**Results:**

A total of 139 participants completed the survey including representation from a range of healthcare professions. A third (36/101, 36%) initiated discussions about gastrostomy later in practice than they believed was ideal. In relation to the outcome of declining compared to accepting gastrostomy, participants were more likely to discuss aspiration (80% vs. 68%), choking (76% vs. 58%) and prognosis (36% vs. 22%). Participants believed gastrostomies should be placed after a mean 8.1% weight loss since symptom-onset. More participants favored gastrostomy placement before pwMND presented with respiratory symptoms (45%) compared to onset of dysphagia (11%). Half believed pwMND placed gastrostomies too late. Participants were more likely to ‘often’/‘always’ recommend pwMND to have a gastrostomy (23%) than continue without (7%) or decline (4%) gastrostomy, when believing these were the best option for pwMND. Nurses and dietitians discussed the broadest range of information, while doctors were more likely to discuss mortality risk and prognosis.

**Conclusion:**

There is variation in HCPs practice and beliefs about initiating discussions, the sharing of information and recommendations, and timing, about gastrostomy placement. The information shared varies by profession and there is evidence of sub-optimal communication between HCPs. Further research is required to understand how these findings may impact on the decision-making of pwMND about gastrostomy.

## Introduction

People living with motor neuron disease (pwMND) face making many decisions throughout their disease course. These decisions are made within the context of experiencing a progressive loss of function, limited treatment options and an average survival of 2–4 years. In the absence of a cure, therapeutic options focus on compensating for functional losses, including dysphagia and respiratory failure (1–3). Between 44% and 85% of pwMND experience dysphagia one year after diagnosis, contributing to a high risk of malnutrition and aspiration (4,5). Malnutrition is an independent prognostic indicator (6,7) and aspiration pneumonia was the reported cause of death in 19% of pwMND (8). Although evidence for improved quality of life or survival remains equivocal (9), gastrostomy placement is routinely offered to pwMND to address the risk of malnutrition or aspiration (10–12).

Guidelines recommend that healthcare professionals (HCPs) collaborate with patients to develop a shared understanding of the benefits and risks of intervention options available and make decisions that are consistent with the patient’s values and preferences (13,14). An emerging qualitative evidence base has captured the contextual factors that influence how, when and why pwMND make decisions about gastrostomy placement (15,16). HCPs are a valued source of information about the disease and interventions for pwMND and can be expected to use their experience and knowledge to guide decision-making (17). Decisions about gastrostomy placement are rarely made between a single HCP and person with MND, in a single interaction. More often, decision-making is distributed over time including interactions with multiple HCPs, caregivers and other sources of information (18–20). A distributed process of decision-making challenges the multidisciplinary team (MDT) to deliver consistent decision support (21–24). There is a paucity of research focusing on the practice and beliefs of different healthcare professions, across key stages of decision-making.

The objectives of this study were to:

1. Understand UK HCPs’ practice and beliefs in relation to supporting pwMND at key stages of decision-making including when discussions about gastrostomy are first initiated, the sharing of information and when decisions about gastrostomy are made.
2. Identify any differences in the practice and beliefs of different professions involved in supporting the decision-making of pwMND.

## Methods

### Study design

A cross-sectional online survey, enabling a broad reach to the different healthcare professions involved in supporting the decision-making of pwMND about gastrostomy placement was chosen as an appropriate study design (25–27). The study report has been guided by the CHERRIES checklist for reporting internet e-surveys (28) (see supplementary information 1).

### Sample and sampling

Four healthcare professions are known to be routinely involved in discussions with pwMND about gastrostomy placement: doctors, dietitians, speech and language therapists (SLTs) and nurses. A snowball sampling strategy was used to recruit a convenience sample, representative of those professions involved in these discussions (29).

### Survey development

The questionnaire was developed using a sequential approach (27,30) including using the findings from a qualitative evidence synthesis (16) and discussion with the research team (AO, VH, CJM), topic experts and a patient panel. A draft survey was refined in response to feedback from 9 HCPs who participated in a pilot to produce a 56-item questionnaire hosted on Qualtrics online survey platform (Qualtrics, Provo, UT) (see Supplementary information 2 for the full questionnaire).

### Data collection

Key gatekeepers, including the MND Association and relevant HCP organizations, agreed to distribute the questionnaire (see supplementary information 3 for a list of gatekeepers) through their communication channels. Reminders were sent every two weeks between 13/6/2022 and 30/8/2022. Participants were asked to read a participant information sheet, answer screening questions, and complete a consent form embedded in the online survey, before proceeding to complete the questionnaire.

### Analysis

Descriptive statistics were used to analyze the data set using IBM SPSS Statistics (Version: 28.0.0.0 (190)). For some findings, response categories have been combined to allow comparisons between related questions. Cross-tabulations were undertaken to identify differences between the responses from doctors, dietitians, SLTs and nurses. The Chi squared test was applied to identify differences in responses for binary outcomes. McNemar’s paired test of symmetry (for binary outcomes) or marginal homogeneity (for ordinal outcomes) were applied to identify differences between the paired responses for related questions.

### Ethics

The study received approval from the University of Sheffield ethics panel (reference: 210151905) and Health Research Authority (HRA) (reference: 308744).

## Results

### Participant characteristics

Of the 212 HCPs confirming they met the inclusion criteria, 139 participants completed the survey including 73 dietitians (53%), 23 nurses (17%), 19 SLTs (14%), 17 doctors (12%), 4 physiotherapists (3%) and 3 occupational therapists (2%). The participants had a mean 11.1 years (SD 8.3, range 1–39) experience of caring for pwMND and the majority were female (123/139, 89%). Participants most frequently (86/138, 62%) responded that <20% of their caseload were pwMND. Discussion about gastrostomy most commonly took place in the homes of pwMND (92/139, 66%), telephone calls (69/139, 50%) and hospital outpatient clinics (60/139, 43%). Participant characteristics are summarized in Table 1 (see supplementary information 4 for a full description of participant characteristics). The following findings present the participant beliefs and practice in relation to supporting pwMND to make decisions about gastrostomy placement (see Supplementary information 5 for the full findings).

**Table 1.** Participant characteristics.

Characteristic	*n* (%)
Gender (*N* = 139)	
Female	123 (89)
Male	14 (10)
Prefer not to say	2 (1)
Healthcare profession (*N* = 139)	
Dietitian	73 (53)
Nurse	23 (17)
Speech and language therapist	19 (14)
Doctor	17 (12)
Physiotherapist	4 (3)
Occupational therapist	3 (2)
Number of pwMND on caseload (*N* = 138)	
0–10	61 (44)
11–20	30 (22)
21–30	16 (12)
31–100	18 (13)
>100	13 (9)
Percentage of clinical caseload that includes pwMND (*N* = 138)	
0–19%	86 (62)
20–39%	22 (16)
40–99%	16 (12)
100%	14 (10)
Number of years experience caring for pwMND (*N* = 83)	Mean 11.1 years (SD 8.3; Range 1–39)
Setting in which discussions about gastrostomy placement occur (*N* = 139)	
Domiciliary visit	92 (66)
Telephone calls	69 (50)
Hospital based out-patient clinic	60 (43)
Hospital ward	59 (42)
Video call	39 (28)
Community based out-patient clinic	36 (26)
Hospice based out-patient clinic	20 (14)
Hospice in-patient	3 (2)
Other	2 (1)

### Stage 1: Initiating discussions about gastrostomy placement with pwMND

Of the participants (115/139, 83%) stating they initiated discussions about gastrostomy placement with pwMND, most were prompted by pwMND presenting with swallowing difficulties (114/115, 99%) and weight loss (105/115, 91%). A similar proportion of participants initiated discussions about gastrostomy placement prior to (42/115, 37%), or following (51/115, 44%) the first presentation of any clinical indications (e.g. dysphagia and weight loss). A third (36/101, 36%) of participants believed they initiated discussions later in practice than was ideal.

### Stage 2: Sharing information with pwMND

#### Outcomes of gastrostomy placement

There were differences with regards to the potential outcomes participants would address with pwMND, when discussing either the option to accept or decline gastrostomy placement (see Table 2). When discussing the potential outcomes of declining gastrostomy placement, participants were more likely to report they discuss the risk of aspiration (80% vs 68%; *p* = 0.018), choking (76% vs. 58%; *p* = 0.0002) and prognosis (36% vs. 22%; *p* = 0.0002). When discussing the potential outcomes of accepting gastrostomy placement, participants were more likely to report they discuss quality of life (83% vs. 75%; *p* = 0.041) and impact on caregivers (63% vs. 50%; *p* = 0.007).

**Table 2.** The outcomes discussed with pwMND when discussing accepting or declining gastrostomy placement.

Outcome of gastrostomy placement discussed	Discussed in relation to accepting gastrostomy (*n* (%)) *N* = 139	Discussed in relation to declining gastrostomy (*n* (%)) *N* = 139	*p*-value
Impact on the person’s weight	111 (80%)	109 (78%)	0.706
Risk of aspiration	95 (68%)	111 (80%)	**0.018**
Risk of choking	80 (58%)	106 (76%)	**0.0002**
Impact on quality of life	115 (83%)	104 (75%)	**0.041**
Time taken to finish meals	98 (71%)	87 (63%)	0.071
Estimated length of life remaining (prognosis)	30 (22%)	50 (36%)	**0.0002**
Impact on caregivers (e.g. family)	87 (63%)	70 (50%)	**0.007**

Note: Significant differences between the proportion of participants who would discuss outcome in relation to accepting and declining gastrostomy indicated in **bold** (McNemar’s paired test of symmetry *p* ⩽ 0.05).

#### Information about gastrostomy placement and life on enteral feeding

When discussing gastrostomy placement, three quarters of participants included details about the procedure (108/139, 77%) and expected length of hospital stay (103/139, 74%). The risk of not surviving the procedure was reported to be discussed by the lowest proportion of participants (54/139, 39%). In relation to life on enteral feeding, enteral feeding methods (119/139, 86%) and the support pwMND will receive from HCPs (118/139, 85%) were discussed by the most participants. Gastrointestinal side-effects were reported as the least discussed (60/139, 43%). There was a varied response in relation to how frequently participants would discuss the option to withdraw enteral feeding in the future at the time of decision-making (never/rarely: 53/139, 38%; sometimes: 43/139, 31%; often/always: 43/139, 31%).

### Stage 3: Making the decision about gastrostomy placement

#### HCP recommendations about gastrostomy placement

Participants reported a varied belief and practice in relation to giving recommendations to pwMND about whether or not to have a gastrostomy placed:

- Half of participants (72/139, 52%) stated they believed HCPs have a responsibility to give pwMND recommendations about whether or not to have a gastrostomy placed.
- Half of participants (71/139, 51%) stated they ‘never’ or ‘rarely’ give pwMND such recommendations in practice.

**Figure 1.**
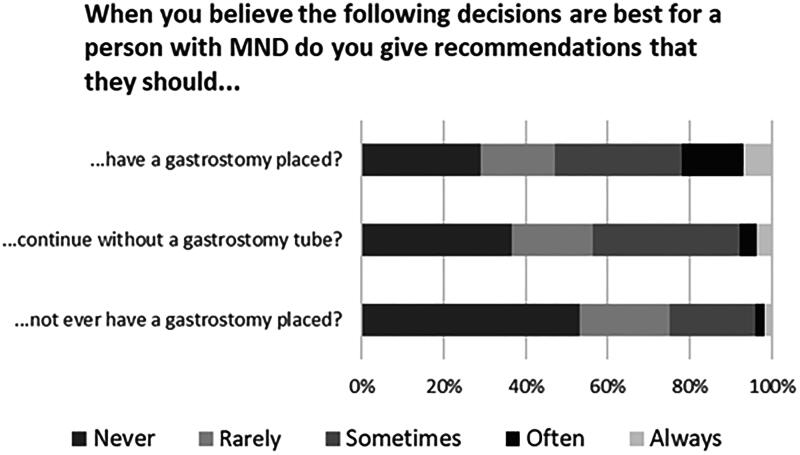
The frequency with which participants would give pwMND recommendations to (1) Have a gastrostomy; (2) Continue without a gastrostomy; (3) Not ever have a gastrostomy placed, when the participant believed that these options were the best course of action for a person with MND to take.

Participants were asked how frequently they would give pwMND a recommendation to: 1. have a gastrostomy placed; 2. continue without a gastrostomy or; 3. never have a gastrostomy; when the participant believed one of these was the best option for a pwMND to take. Participants most commonly responded that they “never” or “rarely” made recommendations but were significantly more likely to state they “often” or “always” recommend pwMND to have a gastrostomy (31/136, 23%) than to continue without (10/137, 7%) or to never have a gastrostomy placed (6/135, 4%) (Figure 1); *p* < 0.001 for all pairwise comparisons.

#### Timing of gastrostomy placement

A similar proportion of participants believed that pwMND should have a gastrostomy placed by the time they have lost 5% (53/124, 43%) or 10% (56/124, 45%) weight compared to their weight at symptom onset (mean 8.1% (SD 3.8; range 1–25%)). Over half (64/124, 52%) reported gastrostomy should be placed when pwMND have lost ≥10% weight since symptom onset.

Participants were asked when they believed pwMND should have a gastrostomy placed in relation to the presentation and severity of respiratory failure and swallowing difficulties. Significantly more participants (*p* < 0.001) believed gastrostomy tubes should be placed earlier in relation to the severity of respiratory symptoms (53/137, 39%; indicated by the cells below the shaded cells in Table 3) than in relation to the severity of dysphagia symptoms (4/137, 3%) (see supplementary information 6 for further information about how categories were combined). A similar proportion of participants believed that pwMND had gastrostomy tubes placed “about the right time” (71/138, 52%) or “too late” (66/138, 48%).

**Table 3.** Participants responses to questions about when they believe pwMND should have a gastrostomy placed in relation to their respiratory and swallowing function.

	When participants believed gastrostomy tubes should be placed in relation to respiratory function (*n* (%))				
When participants believed gastrostomy tubes should be placed in relation to swallowing function (*n*(%))	Prior to any respiratory symptoms	Compensating for early respiratory symptoms, that is, pre-NIV	Experiencing significant consequences, that is, on NIV	Never refer to respiratory failure	Total
Prior to any swallowing problems	13 (10)	1 (1)	0 (0)	1 (1)	15 (11)
Compensating for impact of dysphagia, for example, through texture modified diet	48 (35)	40 (29)	3 (2)	20 (15)	111 (81)
Experiencing significant consequences, for example, chest infections, choking episodes	1 (1)	4 (3)	0 (0)	4 (3)	9 (7)
Never refer to dysphagia	0 (0)	0 (0)	0 (0)	2 (2)	2 (2)
Total	62 (45)	45 (33)	3 (2)	27 (20)	137

### Multidisciplinary decision support

There were differences in the topics discussed with pwMND by different professions. Nurses or dietitians were significantly more likely (*p* ⩽ 0.05) to state they would discuss 17 of the 19 (89%) decision-related information topics, and 12 of the 14 (86%) potential outcomes associated with accepting or declining gastrostomy placement presented as question response options in the survey. In contrast, doctors were significantly more likely (*p* ⩽ 0.05) to state they discuss the impact of respiratory failure on procedural risks (16/17, 94%) and the risks of not surviving the procedure (10/17, 59%), and discuss prognosis in relation to accepting (8/17, 47%) or declining gastrostomy placement (12/17, 71%). See supplementary information 7 for the responses to these questions by each profession.

Most participants (60/138, 44%) believed that information given to pwMND by different members of the MDT was “moderately consistent” and half (72/139, 52%) believed that communication between local HCPs was “very effective.” A third (45/138, 33%) encountered challenges communicating with HCP colleagues. Neurologists (20/40, 50%), gastroenterologists (12/40, 30%) and respiratory doctors (10/40, 25%) were the HCPs that most participants reported a challenge communicating with.

There was a significant difference (*p* = 0.009) between the beliefs of different professions about whether HCPs have a responsibility to give recommendations to pwMND about whether or not to have a gastrostomy, with 74% of nurses (17/23), 65% of doctors (11/17), 45% of dietitians (33/73) and 26% of SLTs (5/19) stating that HCPs should give such recommendations. Significant differences (*p* = 0.008) were similarly identified in relation to reported practice, with 71% of doctors (12/17), 70% of nurses (16/23), 44% of dietitians (32/73) and 21% of SLTs (4/19) “sometimes,” “often” or “always” stating they give pwMND recommendations about whether or not to have a gastrostomy placed.

## Discussion

### Summary of findings

The findings represent a credible account of HCPs’ beliefs and practice in relation to supporting pwMND making decisions about gastrostomy placement. The study identified differences in relation to when discussions about gastrostomy are initiated with pwMND, the information that HCPs share with pwMND and the timing of gastrostomy placement. Nurses and dietitians were more likely to state they address the broadest range of information, except for prognosis and mortality which is more likely to be discussed by doctors. While half of participants believed communication between MDT members was “very effective,” some challenges were identified in relation to communicating with medical professionals.

### Timing of gastrostomy placement

Half of the participants believed that pwMND have gastrostomy tubes placed too late and varied beliefs were expressed about when gastrostomy tubes should be placed in relation to the key indicators of weight loss, dysphagia and respiratory failure. These findings reflect the contention that exists in the literature about the optimal timing of gastrostomy placement (10–12,31,32).

The finding that half of participants believed that gastrostomy tubes should be placed when pwMND have lost >10% weight since symptom onset is consistent with previous case note reviews (33,34). Weight loss after diagnosis and prior to gastrostomy placement is associated with shorter survival informing a recommendation that gastrostomy tubes should be placed prior to pwMND losing 5% weight from diagnosis (7,35). Interestingly, significantly more participants believed pwMND should have a gastrostomy placed earlier in relation to the severity of respiratory failure compared to the severity of dysphagia pwMND are experiencing. While respiratory failure may increase the procedural risks (10,36,37), improving nutritional status and aspiration management are ultimately the primary goals of commencing enteral feeding. The finding that a third of HCPs believe the discussion about gastrostomy is started later than ideal and an acceptance of increased levels of weight loss and dysphagia by the MND MDT may contribute to the delayed placement of a gastrostomy tube and limit the potential for enteral feeding to positively affect the outcomes of pwMND.

There is a need to develop predictive tools that can inform HCPs and pwMND about the outcome of gastrostomy placement in relation to differing presentations of nutritional status, swallowing function and respiratory function. Such decision-support tools could facilitate timely decisions that prevent the development of malnutrition rather than react to it (5,38,39).

### HCP recommendations

Participants’ expressed divided opinions about whether HCPs have a responsibility to give pwMND recommendations about whether or not to have a gastrostomy; a belief reflected in participants’ reported varied use of recommendations in practice. HCPs offering recommendations (40,41) could meet the preferences of some pwMND for HCPs to guide them through the uncertainty of timing of gastrostomy placement (16,17). HCP expertise and opinions are valued by pwMND (17,42,43) and HCP recommendations are often actioned by patients (44). Despite being cited as an essential element of shared decision making (13) concerns remain that decisions informed by HCP recommendations may not be aligned with the values of the patient (45).

A shared understanding of HCPs reasoning (46) for or against gastrostomy placement could help pwMND clarify their own preferences for treatment options available. Interestingly, participants were less likely to state they give pwMND recommendations to continue without or never have a gastrostomy, even when they believed these options were the best for pwMND. These findings suggest an imbalance in how HCPs choose to share their reasoning with pwMND about the best course of action to take. If HCP recommendations are to be used to inform pwMND decision-making, there should be equity in how they are used to aid the understanding of pwMND about all options available including the option to delay or decline gastrostomy placement.

### MND MDT decision support

The survey confirmed the multidisciplinary nature of decision support reported in previous studies (18,19,31,47,48). Significant differences were identified between professions’ responses including nurses and dietitians being more likely to share information about a wider range of issues relevant to the options available, while doctors were more likely to address sensitive issues such as mortality risk or prognosis. Additionally, nurses and doctors were more likely to give pwMND recommendations about whether to have a gastrostomy placed.

These findings suggest that different professions may take on specific responsibilities during the decision-making process (49) and have contrasting views on how to support the decisional needs of pwMND. The reports in qualitative studies, of conflicting information being shared by HCPs (17,22–24) is reflected by 44% of participants believing that their local MND MDT only gives moderately consistent information to pwMND about gastrostomy placement. Inconsistencies in the information shared by different HCPs may lead to decisional conflict and delay the decisions of pwMND about gastrostomy placement, which could, subsequently, impact on patient outcomes.

### Strengths and limitations

The absence of a defined sampling frame limits the generalizability of the findings due to not being able to estimate how representative the sample is of the total population. However, validity is strengthened by the comprehensive sampling strategy employed, using a broad range of gatekeepers to reach HCPs known to be involved in discussions about gastrostomy placement with pwMND. With over half of participants being dietitians (73/139, 53%), there may be some bias toward the dietetic perspective which could impact on the generalizability of the findings.

Further strengths of this study include the rigor with which the survey tool was developed including the conceptualization and design of the survey being informed by the findings of a qualitative evidence synthesis (16,50), collaboration with topic matter experts, and a completion of a pilot study.

### Implications for practice

With a third of participants believing they initiate discussions about gastrostomy later in practice than is ideal, MND MDTs should aim to identify and address any barriers to introducing the intervention to pwMND. Professional guidance recommending gastrostomy placement is discussed ‘early’ is vague and lacks concrete guidance about how and when to open these sensitive conversations. Providing psychological support for pwMND to engage in discussions may allow pwMND more time to deliberate about their options and ultimately lead to more timely commencement of the intervention. HCP recommendations may help pwMND navigate the uncertainty in relation to the timing of gastrostomy placement. HCPs should carefully consider how they communicate their preferences for starting gastrostomy feeding, to allow pwMND to make informed decisions that remain aligned with their own values.

## Conclusion

The findings of this survey have highlighted differences in the beliefs and practice of the different HCPs’ involved in discussions with pwMND about gastrostomy placement. Any delay in initiating the discussion and beliefs about how progression of indicators inform need for gastrostomy, may contribute to the perceived late placement of gastrostomy tubes. MND services should seek to develop decision-support care pathways, including the range of HCPs and teams involved in these discussions with pwMND, that aim to improve lines of communication and enable a consistent approach to supporting the decision-making of pwMND. Such MDT decision-support frameworks should account for the variation in information and professional recommendations shared with pwMND by individual HCPs. Further research is required to understand the HCP, pwMND and organizational barriers to initiating earlier discussions and to the timely placement of gastrostomy tubes in those pwMND who wish to proceed with the intervention.

## Supplementary Material

Supplemental Material

## Data Availability

The authors confirm that the data supporting the findings of this study are available within the article and its supplementary materials.
