# Supplementary material for: Supporting people with Motor Neuron Disease (MND) to make decisions about gastrostomy feeding tube placement: a survey of UK healthcare professionals’ practice and beliefs
Source: Amyotroph Lateral Scler Frontotemporal Degener. 2024 Feb 9;25(3-4):290–8. doi: 10.1080/21678421.2024.2314061 (PMC11262427; doi:10.1080/21678421.2024.2314061)
Supplement: Supplemental Material [file IAFD_A_2314061_SM4878.docx]

**Supplementary Information**

**Supplementary information 1.**

Evidence for how the study met the requirements of the CHERRIES checklist (Eysenbach 2004).

| ***Item category*** | ***Checklist item*** | ***Explanation*** |
| --- | --- | --- |
| **Design** | Describe survey design | A rigorous approach was taken to developing and testing the survey prior to launch.  Target population clearly identified as any UK HCP who supports pwMND to make decisions about gastrostomy placement.  In the absence of a defined sampling frame a convenience sample was obtained using a snowball sampling strategy. |
| **Ethics** | Ethics board approval | Ethical approval was granted by the University of Sheffield ethics board and approval also given by HRA |
|  | Informed consent | The participant information sheet (PIS), screening questions and consent form were embedded into the online survey. The PIS included information about the study, how long it would take to complete and how their data would be used. Potential participants were required to complete a screening question to confirm they met the inclusion criteria for the study. Finally, the participants were required to confirm they agreed with statements within a consent form prior to entering the survey. Participants were made aware that only submitted surveys would be included in the analysis. |
|  | Data protection | No participant identifiable information was collected within the survey. |
| **Development and pre-testing** | Development and testing | The draft survey instrument was uploaded onto Qualtrics and piloted with 9 HCPs from the target population. The pilot participants completed a structured feedback form which informed the final changes made to the survey prior to launch. |
| **Recruitment process and description of the sample having access to the questionnaire** | Open survey versus closed survey | The survey was open to anyone who had the link to the Qualtrics survey. |
|  | Contact mode | A snowball sampling strategy was used. This included gatekeeper organisations and groups forwarding an email including a brief summary of the study and the link to the survey to their members. The gatekeepers and lead researcher also shared the survey link using social media. |
|  | Advertising the survey | The link to the survey was shared by gatekeepers and the lead researcher via email and social media. The MND Association, British Association of Parenteral and Enteral Nutrition (BAPEN) and the Parenteral and Enteral Specialist Group of the British Dietetic Association (PENG) also shared the link to the survey on their websites. |
| **Survey administration** | Web/email | Qualtrics online survey software was used to host the survey. |
|  | Context | The link to the survey was disseminated via email and social media, and via news stories on MND Association, BAPEN and PENG websites. |
|  | Mandatory/voluntary | Potential participants were made explicitly aware that it was completely voluntary for them to complete the survey. Participants did not have to answer every question. |
|  | Incentives | No incentives were offered to complete the survey. |
|  | Time/date | The original plan was for the survey to remain open for 8 weeks. However, the closing date was extended by 2 weeks as the target sample size had not been achieved by the end of the 8 weeks. The survey was open between 13/6/2022 and 30/8/2022. |
|  | Randomisation of items or questionnaires | No randomisation of items or questionnaires was used in this survey. |
|  | Adaptive questioning | Some conditional branching was used within the survey to only show certain questions dependent on answers to previous questions (e.g. questions about initiating discussions with pwMND about gastrostomy were only displayed to those participants who stated start such discussions). The main purpose of this was to reduce the number of questions being asked and to support validity of the study. |
|  | Number of items | Only one question was asked per page of the survey. The survey included a maximum of 56 questions in total. |
|  | Number of screens (pages) | The survey was distributed over 56 pages (screens). |
|  | Completeness check | The survey did not include any completeness check allowing participants to not complete questions if they did not wish to rather than including an option to state they do not wish to answer the question. Participants were made aware in the PIS that they did not have to provide a response to every question. No questions were mandatory. |
|  | Review step | Respondents were able to move backwards through their survey and alter their responses if they wished to. |
| **Response rates** | Unique site visitor | No steps were made to determine if participants completing the survey were unique. This would raise the risk of the same participant completing the survey more than once. |
|  | View rate | Unique site visitors not recorded |
|  | Participation rate | The participants were required to reach the end of the survey and submit their survey to be included in the study |
|  | Completion rate | 139 participants submitted the survey out of a total of 212 who confirmed that they met the inclusion criteria for the study. |
| **Preventing multiple entries from the same individual** |  | No steps were taken to ensure that multiple entries were recorded from the same individual. |
| **Analysis** | Handling of incomplete questionnaires | Only questionnaires that were submitted by the participant were included in the analysis. Within the reporting it is made clear how many participants answered each question. |
|  | Questionnaires submitted with an atypical timestamp | No timeframe was implemented for which participants should take to complete the questionnaire. |
|  | Statistical correction | No weighting or propensity scores were used to adjust for a non-representative sample. |

**Supplementary information 2**

**Survey proforma. See below for a printed version of the questionnaire that was uploaded onto the Qualtrics online survey platform.**

The survey is divided into 4 sections: The initiation of discussions about gastrostomy placement with people diagnosed with MND The process of making the decision about having a gastrostomy placed How your local care team collaborate to support people with MND considering gastrostomy placement Demographic questions about you and your professional experience (your answers to these questions are really important to allow the survey results to be analysed)

The following questions focus on when healthcare professionals**first introduce**gastrostomy feeding tube placement to people with MND.

Do you initiate discussions about gastrostomy placement with people with MND?

- Yes (1)
- No (2)

Which of the following factors prompt you to initiate discussions about gastrostomy placement with people with MND? (tick all that apply)

- Weight loss (1)
- Low body weight (9)
- Swallowing difficulties (2)
- Respiratory failure (3)
- Poor appetite (4)
- Dehydration (7)
- Poor lip seal (5)
- Upper limb weakness (6)
- Lower limb weakness (10)
- Prolonged mealtimes (12)
- Time and effort to prepare meals (13)
- Caregivers needing to assist people with MND to eat and drink (14)
- Difficulties taking medications (15)
- Person with MND requesting information on gastrostomy placement (16)
- Caregiver requesting information on gastrostomy placement (17)
- Other (8) ________________________________________________

In most cases, **WHEN DO YOU** initiate discussions about gastrostomy placement with people with MND? (tick one option only)

- Prior to the onset of any indications for gastrostomy (1)
- Following the **first** presentation of **any** indications for gastrostomy (2)
- When there is evidence that the indications for gastrostomy are progressing (3)
- Other (please state) (4) ________________________________________________

In most cases, when do you think gastrostomy placement **SHOULD**be first discussed with people with MND? (tick one option only)

- Prior to the onset of any indications for gastrostomy (1)
- Following the **first** presentation of **any** indications for gastrostomy (2)
- When there is evidence of the indications for gastrostomy are progressing (3)
- Other (please state) (4) ________________________________________________

Do you have any local policies, care pathways or guidance that guides healthcare professionals about when to initiate discussions about gastrostomy placement to people with MND?

- Yes (1)
- No (2)

If you would you be happy to share your local guidance with the us, please email an electronic copy to smwhite1@sheffield.ac.uk (please note that your answers to the survey will remain anonymous)

The following questions focus on the process of making decisions about gastrostomy placement with people with MND.

Which of the following options do you routinely present to people with MND when discussing gastrostomy placement? (tick all that apply)

- They can have a gastrostomy placed (1)
- They can continue without a gastrostomy but can still choose to have a gastrostomy at a later date (2)
- They can decline to have a gastrostomy placed ever (3)
- I do not present any options (4)
- Other (please specify) (5)

Which aspects of the procedure to place a gastrostomy do you routinely discuss with people with MND? (tick all that apply)

- The impact of respiratory failure on the risks associated with the procedure to place a gastrostomy (1)
- Detail about the procedure to place the gastrostomy (10)
- The sedation required during the procedure (11)
- Length of hospital stay (3)
- Risk of aspiration (4)
- Risk of not surviving gastrostomy placement (5)
- Pain post gastrostomy placement (6)
- Risk of infection post gastrostomy placement (7)
- Other (8) ________________________________________________
- I do not discuss the procedure to place a gastrostomy with people with MND (9)

Which of the following issues relating to living with a gastrostomy tube do you routinely discuss with people with MND? (tick all that apply)

- Care of the gastrostomy (1)
- Care of the gastrostomy stoma site (2)
- Impact gastrostomy may have on body image (3)
- Later presenting tube complications and how they are managed e.g. blocked, damaged or displaced tubes (4)
- Later presenting stoma complications and how they are managed e.g. infection, overgranulation or leakage (14)
- Gastrointestinal side effects of gastrostomy feeding e.g. diarrhoea, constipation, bloating (5)
- The healthcare professional support they will receive once home with gastrostomy tube (7)
- Enteral feed methods available e.g. bolus and pump feeding (8)
- The impact gastrostomy feeding will have on caregivers (9)
- Identifying who will use and care for the tube post placement (10)
- The option to withdraw enteral feeding in the future (11)
- Other (12) ________________________________________________
- I do not discuss living with a gastrostomy tube with people with MND (13)

Which outcomes of **COMMENCING** gastrostomy feeding do you routinely discuss with people with MND? (tick all that apply)

- Impact on the person's weight (1)
- Risk of aspiration (2)
- Risk of choking (3)
- Impact on quality of life (4)
- Time taken to finish meals (8)
- Estimated length of life remaining (prognosis) (5)
- Impact on caregivers (e.g. family) (9)
- Other (6) ________________________________________________
- I do not discuss outcomes of commencing gastrostomy feeding with people with MND (7)

Which outcomes of **DECLINING**gastrostomy feeding do you routinely discuss with people with MND? (tick all that apply)

- Impact on the person's weight (1)
- Risk of aspiration (2)
- Risk of choking (3)
- Impact on quality of life (4)
- Time taken to finish meals (8)
- Estimated length of life remaining (prognosis) (5)
- Impact on caregivers (e.g. family) (9)
- Other (6) ________________________________________________
- I do not discuss outcomes of not commencing gastrostomy feeding with people with MND (7)

What goals of gastrostomy feeding do you typically discuss with people with MND during decision making? (tick all that apply)

- Weight maintenance/increase (1)
- Reduce risk of aspiration (2)
- Prevent choking (3)
- Provide an alternative route for medication administration (4)
- Reduce fatigue (5)
- Prolong life (6)
- Prevent/slow muscle wasting (7)
- Improve quality of life (8)
- Reduce caregiver burden (9)
- Improve caregivers quality of life (10)
- Reduce stress/burden at mealtimes for person with MND (11)
- Other (12) ________________________________________________

How long do you tell people with MND they will typically have to wait to have a gastrostomy placed following referral? (tick one option only)

- 1 week (1)
- 2 weeks (2)
- 3 weeks (3)
- 4 weeks (4)
- 5 weeks (5)
- 6 weeks (6)
- 7 weeks (7)
- 8 weeks (8)
- If more than 8 weeks please state (9) ________________________________________________
- I do not inform people with MND about how long they will have to wait to have the gastrostomy placed (10)

What is the average period of time you inform people with MND they will be in hospital for while having their gastrostomy placed? (tick one option only)

- 0 days (i.e. gastrostomy placed as a day-case) (1)
- 1 day (2)
- 2 days (3)
- 3 days (4)
- 4 days (5)
- 5 days (6)
- 6 days (7)
- 7 days (8)
- >7 days (please state average number of days you give) (9) ________________________________________________
- I do not discuss length of hospital stay (10)

What information sources about the decision to have a gastrostomy placed do you direct people with MND to? (tick all that apply)

- Locally produced patient information leaflet about gastrostomy placement (1)
- Locally produced patient information leaflet about home enteral feeding (2)
- Mytube website (3)
- MND Association eating and drinking guide (4)
- MND Association tube feeding leaflet (5)
- Parenteral and Enteral Nutrition Specialist Group of the British Dietetic Association (PENG) enteral feeding leaflet: ‘Enteral tube feeding – your questions answered’ (6)
- PENG leaflet: ‘Having an enteral feeding tube – further questions to ask (7)
- Patients on Intravenous and Naso-gastric Nutrition Treatment (PINNT) (8)
- I do not provide any information resources (9)
- Other (10) ________________________________________________

Do you practically demonstrate the following equipment to people with MND when discussing gastrostomy placement? (tick all that apply)

- The gastrostomy tube (1)
- Enteral feeding syringes (2)
- Enteral feeding pump, stand and giving sets (3)
- Other (please specify) (4) ________________________________________________
- I do not demonstrate any of the above equipment (5)
- I refer to another healthcare professional or service to demonstrate the equipment to people with MND (please specify the professional you refer the patient to) (6) ________________________________________________

How often do you encourage people with MND considering gastrostomy placement to meet other people with MND?

- Never (6)
- Rarely (7)
- Sometimes (8)
- Often (9)
- Always (10)

The following questions focus on healthcare professionals' recommendations to people with MND. For the purposes of these questions, 'recommendations' are defined as:
**‘when healthcare professionals give opinions to people with MND about whether or not they should have a gastrostomy placed.’**

In your opinion, do healthcare professionals have a responsibility to give recommendations to people with MND about whether or not to have a gastrostomy placed? (tick one option only)

- Yes (1)
- No (2)

Do you give recommendations to people with MND about whether or not they should have a gastrostomy placed? (tick one option only)

- Never (35)
- Rarely (36)
- Sometimes (37)
- Often (38)
- Always (39)

Do people with MND ask you to give a recommendation about whether or not they should have a gastrostomy tube placed? (tick one option only)

- Never (11)
- Rarely (12)
- Sometimes (13)
- Often (14)
- Always (15)

When you believe the following decisions are best for a person with MND do you give recommendations that they should...

|  | Never (6) | Rarely (7) | Sometimes (8) | Often (9) | Always (10) |
| --- | --- | --- | --- | --- | --- |
| ...have a gastrostomy placed? (1) |  |  |  |  |  |
| ...continue without a gastrostomy tube? (2) |  |  |  |  |  |
| ...not ever have a gastrostomy placed? (3) |  |  |  |  |  |

Do you discuss the future withdrawal of enteral feeding during decision making discussions about gastrostomy placement? (tick on option only)

- Never (11)
- Rarely (12)
- Sometimes (13)
- Often (14)
- Always (15)

Have you attended training or study events on the following topics? (tick all that apply)

- MND disease course (1)
- Supporting people making decisions about interventions (3)
- Nutritional management of people with MND (4)
- Gastrostomy placement procedures (5)
- Communication skills (6)

On average how long will you typically spend discussing gastrostomy placement with a person with MND? (tick one option only)

- 0-15 mins (1)
- 16-30 mins (2)
- 31-45 mins (3)
- 46-60 mins (4)
- >60 mins (5)

Do you feel you have enough time to discuss gastrostomy placement during consultations with people with MND? (tick one option only)

- Yes (3)
- No (4)

Please explain why you feel you do not have enough time to discuss gastrostomy placement during consultations with people with MND

________________________________________________________________

________________________________________________________________

________________________________________________________________

________________________________________________________________

________________________________________________________________

The following 3 questions relate to the timing of gastrostomy placement i.e. when you feel people with MND **should have had a gastrostomy tube placed by**, in relation to their weight, respiratory function and swallowing ability. Please assume when answering these questions that the person with MND has agreed to gastrostomy placement and is now deciding **WHEN** they should have it placed.

Ideally, by **what percentage (%) weight loss from symptom onset** do you feel people with MND should have had a gastrostomy placed? (choose from drop down list)

▼ 1 (1) ... 100 (100)

Ideally, in relation to their respiratory function, when do you feel people with MND should have a gastrostomy placed? (tick one option only)

- Prior to presenting with any respiratory symptoms or objective signs of respiratory failure (1)
- When they have symptoms of respiratory failure but objective respiratory measures remain in normal range (2)
- When they have symptoms of respiratory failure and objective respiratory measures are outside normal range (3)
- When initiated on non-invasive ventilation (NIV) (4)
- When they are using non-invasive ventilation (NIV) for more than 8 hours per 24 hour period (5)
- When they are using non-invasive ventilation (NIV) for more than 16 hours per 24 hour period (6)
- I do not refer to markers of respiratory failure when making decisions regarding gastrostomy insertion (7) Q128 Ideally, in relation to their swallowing function, when do you feel people with MND should have a gastrostomy placed? (tick one option only)
- Prior to presenting with any swallowing difficulties (1)
- When they are noticing swallowing difficulty but not needing to modify the texture of their diet or fluids (2)
- When they need to modify the texture of diet or fluids but continue to manage adequate amounts (3)
- When they are presenting with adverse consequences of swallowing difficulties e.g. chest infections, choking episodes, reduced intake, prolonged mealtimes (4)
- When the consequences of swallowing difficulties present a significant risk to the respiratory health or nutritional intake of the person with MND (5)
- I do not refer to markers of swallow function when making decisions (6)

In your opinion do most people with MND agree to gastrostomy placement:

- Too early (1)
- About the right time (2)
- Too late (3)

In your opinion, to what extent are people with MND placed under pressure to agree to gastrostomy placement by:

|  | Never (11) | Rarely (12) | Sometimes (13) | Often (14) | Always (15) |
| --- | --- | --- | --- | --- | --- |
| Healthcare professionals (1) |  |  |  |  |  |
| Family members (2) |  |  |  |  |  |

The following question focuses on how different healthcare professionals work together to support people with MND making decisions about gastrostomy placement. When questions state **'in your local area'**, this refers to the other healthcare professionals who are involved in discussions with people with MND that you also support including those in community teams and MND clinics.

Which healthcare professionals are involved in supporting people with MND to make decisions about gastrostomy placement in your local area (tick all that apply)?

- Neurologist (1)
- Palliative care doctor (2)
- Palliative care nurse (3)
- Gastroenterologist (4)
- Radiologist (5)
- Anaesthetist (6)
- Respiratory doctor (7)
- Respiratory physiotherapist (11)
- Respiratory nurse (21)
- Physiotherapist (non-respiratory specialist) (18)
- MND/neurology Specialist Nurse (8)
- MND coordinator (19)
- Community neurology matron (22)
- Home Enteral Feed Dietitian (10)
- Dietitian (non-home enteral feeding) (9)
- Speech and language therapist (12)
- Nutrition/gastrostomy nurse or advanced clinical practitioner (13)
- Occupational therapist (15)
- Psychologist (16)
- Social worker (17)
- General practitioner (GP) (14)
- Other (please specify) (20) ________________________________________________

What guidance do you refer to for supporting people with MND to make decisions about gastrostomy placement? (tick all that apply)

- NICE MND guideline [NG42] (1)
- European Federation of Neurological Societies (EFNS) guidelines (2)
- American Academy of Neurology (AAN) ‘Care of patient with ALS’ guideline (3)
- NICE nutrition support clinical guideline [CG32] (4)
- ESPEN guideline clinical nutrition in neurology (5)
- NICE shared decision making guideline [NG197] (6)
- Locally developed guideline (7)
- Other (please specify) (8) ________________________________________________
- I do not refer to any guidance (14)

If you would you be happy to share your locally developed guideline with the us, please email an electronic copy to smwhite1@sheffield.ac.uk (please note that your answers to the survey will remain anonymous)

How useful is the available guidance at informing your practice when supporting people with MND to make decisions about gastrostomy placement? (tick one option only)

- Extremely useful (1)
- Very useful (2)
- Moderately useful (3)
- Slightly useful (4)
- Not at all useful (5)

How often are you aware of the discussions other healthcare professionals have had with people with MND about gastrostomy placement? (tick one option only)

- Never (6)
- Rarely (7)
- Sometimes (8)
- Often (9)
- Always (10)

Do you communicate with other healthcare professionals **AFTER** having  discussions with people with MND about gastrostomy placement? (tick one option only)

- Never (10)
- Rarely (11)
- Sometimes (12)
- Often (13)
- Always (14)

How do you communicate with other healthcare professionals? (tick all that apply)

- Letter (1)
- Email (2)
- Telephone (3)
- In MDT meetings (4)
- Via message function in electronic patient records (5)
- Other (6) ________________________________________________
- I do not communicate with other healthcare professionals after discussions with people with MND about gastrostomy placement (7)

In your opinion, how important is it for healthcare professionals to communicate with other members of the care team **AFTER** they have had discussions about gastrostomy placement with people with MND? (tick one option only)

- Extremely important (1)
- Very important (2)
- Moderately important (3)
- Slightly important (4)
- Not at all important (5)

What communication do you send to the person with MND following discussions about gastrostomy placement? (tick all that apply)

- Copy person with MND into letter sent to healthcare professionals (1)
- Copy person with MND into emails sent to healthcare professionals (2)
- Send individual letter or email to person with MND summarising content of discussion and decisions made (3)
- Update a patient held record (4)
- I do not communicate with the person with MND outside of direct contact e.g. clinic, home visit, telephone or video call (5)
- Other (6) ________________________________________________

Do you encounter any challenges communicating with any members of the care team in your local area when supporting people with MND to make decisions about gastrostomy placement?

- Yes (85)
- No (86)

Do you experience these challenges specifically with any of the following professionals? (tick all that apply)

- Neurologist (1)
- Palliative care doctor (2)
- Palliative care nurse (3)
- Gastroenterologist (4)
- Radiologist (5)
- Anaesthetist (6)
- Respiratory doctor (7)
- Respiratory Physiotherapist (11)
- Respiratory nurse (21)
- Physiotherapist (non-respiratory specialist) (22)
- MND/neurology Specialist Nurse (8)
- MND coordinator (23)
- Community neurology matron (24)
- Home Enteral Feed Dietitian (10)
- Dietitian (non-home enteral feeding) (9)
- Speech and language therapist (12)
- Nutrition/gastrostomy nurse or advanced clinical practitioner (13)
- Occupational therapist (15)
- Psychologist (16)
- Social worker (17)
- General Practitioner (GP) (19)
- Other (please specify) (20) ________________________________________________

Please describe any challenges you experience.

________________________________________________________________

________________________________________________________________

________________________________________________________________

________________________________________________________________

________________________________________________________________

How effective do you believe the communication is between healthcare professionals in your local area when supporting people with MND to make decisions about gastrostomy placement? (tick one option only)

- Not effective at all (1)
- Slightly effective (2)
- Moderately effective (3)
- Very effective (4)
- Extremely effective (6)

Do you attend meetings (e.g. multidisciplinary team (MDT) meetings) with other healthcare professionals where you discuss people with MND making decisions about gastrostomy? (tick one option only)

- Yes (3)
- No (4)

Are there any professionals or professional teams that you feel are missing from these meetings who are involved in discussions about gastrostomy placement? (tick one option only)

- Yes (3)
- No (4)

In your opinion which professionals or teams are missing from your MDT meetings? (tick all that apply)

- Neurologist (1)
- Palliative care team (2)
- Gastrostomy placement team (4)
- Anaesthetist (6)
- Respiratory team (7)
- MND/neurology Specialist Nurse or coordinator (8)
- Home Enteral Feed Team (10)
- Dietitian (non-home enteral feeding) (13)
- Physiotherapist (non-respiratory specialist) (11)
- Speech and language therapist (12)
- Enteral feeding advanced clinical practitioner (14)
- Occupational therapist (15)
- Psychologist (16)
- Social worker (17)
- General practitioner (GP) (19)
- Ward nurse (18)
- Other (please specify) (20) ________________________________________________

How often do the MDT meetings take place? (tick one option only)

- Weekly (1)
- Every two weeks (2)
- Every 3 weeks (3)
- Every month (4)
- Every 2 months (5)
- Every 3 months (6)
- Other (please specify) (7) ________________________________________________

In your opinion, how consistent is the information given to people with MND about gastrostomy placement, by different members of your local MND care team?

- Extremely consistent (1)
- Very consistent (2)
- Moderately consistent (3)
- Slightly consistent (4)
- Not at all consistent (5)

What is your sex?

- Male (1)
- Female (2)
- Non-binary / third gender (3)
- Prefer not to say (4)
- Other (please specify) (5) ________________________________________________

What is your profession? (tick one option only)

- Doctor (1)
- Nurse (2)
- Dietitian (3)
- Speech and language therapist (4)
- Occupational therapist (5)
- Physiotherapist (6)
- Social worker (7)
- Psychologist (8)
- Other (9) ________________________________________________

What is your professional role?

- Neurologist (1)
- Palliative care doctor (2)
- Palliative care nurse (3)
- Gastroenterologist (4)
- Radiologist (5)
- Anaesthetist (6)
- Respiratory doctor (7)
- Respiratory physiotherapist (11)
- Respiratory nurse (21)
- Physiotherapist (non-respiratory specialist) (22)
- MND/neurology Specialist Nurse (8)
- MND coordinator (23)
- Community neurology matron (24)
- Home Enteral Feed Dietitian (10)
- Dietitian (non-home enteral feeding) (9)
- Speech and language therapist (12)
- Nutrition/gastrostomy nurse (13)
- Enteral feeding advanced clinical practitioner (14)
- Occupational therapist (15)
- Psychologist (16)
- Social worker (17)
- Ward nurse (18)
- Other (please specify) (20) ________________________________________________
- General practitioner (GP) (19)

During a typical working week, how much of your time involves supporting people with MND? (tick one option only)

- None of your time (1)
- Very little of your time (2)
- Some of your time (3)
- The majority of your time (4)
- All of your time (5)

Approximately, how many people with MND do you currently have on your caseload? (tick one option only)

- 0-10 (1)
- 11-20 (2)
- 21-30 (3)
- 31-40 (4)
- 41-50 (5)
- 51-60 (6)
- 61-70 (7)
- 71-80 (8)
- 81-90 (9)
- 91-100 (10)
- >100 (11)

What percentage of your current clinical caseload is people with MND?

- 0-19% (1)
- 20-39% (2)
- 40-59% (3)
- 60-79% (4)
- 80-99% (5)
- 100% (6)

How many years experience do you have caring for people with MND? (choose number from drop down box)

▼ 1 (1) ... >60 (61)

In what setting do you see people with MND when having discussions about gastrostomy placement? (tick all that apply)

- Hospital ward (1)
- Hospital based out-patient clinic (2)
- Hospice based out-patient clinic (3)
- Community based out-patient clinic (4)
- Domiciliary visit (5)
- Telephone calls (6)
- Video call (7)
- Other (8) ________________________________________________

We are planning a subsequent study that will involve observing how people with MND are supported to make decisions about gastrostomy placement by their healthcare professionals, including interviews of people with MND, caregivers and healthcare professionals. If you are interested in being involved in this study please email the lead researcher at smwhite1@sheffield.ac.uk.

By clicking on the 'Submit' button, your survey will be submitted and you will no longer be able to change your answers.

**Supplementary information 3.**

**List of gatekeepers for distributing the survey link.**

Below is a list of the organisations who assisted in disseminating the link to their members who may have met the inclusion criteria for the study:

- MND Clinical Studies Group (MNDCSG)
- Association of palliative medicine
- British Association of parenteral and enteral nutrition (BAPEN)
- British Dietetic Association (BDA)
- Parenteral and enteral specialist group of the BDA (PENG)
- Neurosciences specialist group of the BDA (NSG)
- PENG Virtual HEF Group
- MND Association
- National Nurse Nutrition Group (NNNG)

**Supplementary information 4.**

**Characteristics and experience of survey participants.**

| **Question** | N (%) |
| --- | --- |
| **What is your sex?** (n=139) |  |
| Male | 14 (10) |
| Female | 123 (89) |
| Prefer not to say | 2 (1) |
| Other (please specify) | 0 (0) |
| **What is your profession?** (n=139) |  |
| Doctor | 17 (12) |
| Nurse | 23 (17) |
| Dietitian | 73 (53) |
| Speech and language therapist | 19 (14) |
| Occupational therapist | 3 (2) |
| Physiotherapist | 4 (3) |
| **What is your professional role?** (n=138) |  |
| Neurologist | 4 (3) |
| Palliative care doctor | 7 (5) |
| Gastroenterologist | 2 (1) |
| Respiratory doctor | 4 (3) |
| Respiratory physiotherapist | 1 (1) |
| Respiratory nurse | 1 (1) |
| Physiotherapist (non-respiratory specialist) | 3 (2) |
| MND/neurology Specialist Nurse | 12 (9) |
| MND coordinator | 2 (1) |
| Home Enteral Feed Dietitian | 42 (30) |
| Dietitian (non-home enteral feeding) | 30 (22) |
| Speech and language therapist | 18 (13) |
| Nutrition/gastrostomy nurse | 8 (6) |
| Enteral feeding advanced clinical practitioner | 2 (1) |
| Occupational therapist | 2 (1) |
| **During a typical working week, how much of your time involves  supporting people with MND?** (n=139) |  |
| None of your time | 3 (2) |
| Very little of your time | 19 (14) |
| Some of your time | 88 (63) |
| The majority of your time | 14 (10) |
| All of your time | 15 (11) |
| **Approximately, how many people with MND do you currently have on your caseload?** (n=138) |  |
| 0-10 | 61 (44) |
| 11-20 | 30 (22) |
| 21-30 | 16 (12) |
| 31-40 | 6 (4) |
| 41-50 | 2 (1) |
| 51-60 | 3 (2) |
| 61-70 | 0 (0) |
| 71-80 | 3 (2) |
| 81-90 | 2 (1) |
| 91-100 | 2 (1) |
| >100 | 13 (9) |
| **What percentage of your current clinical caseload is people with MND?** (n=138) |  |
| 0-19% | 86 (62) |
| 20-39% | 22 (16) |
| 40-59% | 10 (7) |
| 60-79% | 1 (1) |
| 80-99% | 5 (4) |
| 100% | 14 (10) |
| **How many years experience do you have caring for people with MND?** (n=83) |  |
| Mean 11.1 years (SD 8.3)  Range 1-39 year |  |
| **In what setting do you see people with MND when having discussions about gastrostomy placement?** (n=139) |  |
| Hospital ward | 59 (42) |
| Hospital based out-patient clinic | 60 (43) |
| Hospice in-patient | 3 (2) |
| Hospice based out-patient clinic | 20 (14) |
| Community based out-patient clinic | 36 (26) |
| Domiciliary visit | 92 (66) |
| Telephone calls | 69 (50) |
| Video call | 39 (28) |
| Other | 2 (1) |

**Supplementary information 5.**

**Table 3.** The full data set.

| Question | n (%) |
| --- | --- |
| **Do you initiate discussions about gastrostomy placement with people with MND?** (N=139) |  |
| Yes | 115 (83) |
| No | 24 (17) |
| **Which of the following factors prompt you to initiate discussions about gastrostomy placement with people with MND?** (N=115) |  |
| Swallowing difficulties | 114 (99) |
| Weight loss | 105 (91) |
| Person with MND requesting information on gastrostomy placement | 103 (90) |
| Difficulties taking medications | 94 (82) |
| Caregiver requesting information on gastrostomy placement | 93 (81) |
| Dehydration | 89 (77) |
| Poor appetite | 80 (70) |
| Prolonged mealtimes | 81 (70) |
| Low body weight | 76 (66) |
| Respiratory failure | 72 (63) |
| Poor lip seal | 51 (44) |
| Time and effort to prepare meals | 49 (43) |
| Caregivers needing to assist people with MND to eat and drink | 45 (39) |
| Upper limb weakness | 37 (32) |
| Lower limb weakness | 10 (9) |
| Other | 20 (18) |
| **In most cases, WHEN DO YOU initiate discussions about gastrostomy placement with people with MND?** (N=115) |  |
| Prior to the onset of any indications for gastrostomy | 42 (37) |
| Following the first presentation of any indications for gastrostomy | 51 (44) |
| When there is evidence that the indications for gastrostomy are progressing | 15 (13) |
| Other | 7 (6) |
| **In most cases, when do you think gastrostomy placement SHOULD be first discussed with people with MND?** (N=137) |  |
| Prior to the onset of any indications for gastrostomy | 91 (66) |
| Following the first presentation of any indications for gastrostomy | 33 (24) |
| When there is evidence of the indications for gastrostomy are progressing | 5 (4) |
| Other | 8 (6) |
| **Which aspects of the procedure to place a gastrostomy do you routinely discuss with people with MND?** (N=139) |  |
| Detail about the procedure to place the gastrostomy | 108 (77) |
| Length of hospital stay | 103 (74) |
| The impact of respiratory failure on the risks associated with the procedure to place a gastrostomy | 97 (70) |
| Risk of infection post gastrostomy placement | 89 (64) |
| Pain post gastrostomy placement | 88 (63) |
| Risk of aspiration | 83 (60) |
| The sedation required during the procedure | 79 (57) |
| Risk of not surviving gastrostomy placement | 54 (39) |
| I do not discuss the procedure to place a gastrostomy with people with MND | 10 (7) |
| Other | 31 (22) |
| **Which of the following issues relating to living with a gastrostomy tube do you routinely discuss with people with MND?** (N=139) |  |
| Enteral feed methods available e.g. bolus and pump feeding | 119 (86) |
| The healthcare professional support they will receive once home with gastrostomy tube | 118 (85) |
| Identifying who will use and care for the tube post placement | 112 (81) |
| Care of the gastrostomy | 105 (76) |
| The impact gastrostomy feeding will have on caregivers | 91 (66) |
| Care of the gastrostomy stoma site | 90 (65) |
| Later presenting tube complications and how they are managed e.g. blocked, damaged or displaced tubes | 80 (58) |
| The option to withdraw enteral feeding in the future | 79 (57) |
| Later presenting stoma complications and how they are managed e.g. infection, overgranulation or leakage | 68 (49) |
| Impact gastrostomy may have on body image | 63 (45) |
| Gastrointestinal side effects of gastrostomy feeding e.g. diarrhoea, constipation, bloating | 60 (43) |
| I do not discuss living with a gastrostomy tube with people with MND | 10 (7) |
| Other | 12 (9) |
| **Which outcomes of COMMENCING gastrostomy feeding do you routinely discuss with people with MND?** (N=139) |  |
| Impact on quality of life | 115 (83) |
| Impact on the person's weight | 111 (80) |
| Time taken to finish meals | 98 (71) |
| Risk of aspiration | 95 (68) |
| Impact on caregivers (e.g. family) | 87 (63) |
| Risk of choking | 80 (58) |
| Estimated length of life remaining (prognosis) | 30 (22) |
| I do not discuss outcomes of commencing gastrostomy feeding with people with MND | 5 (4) |
| Other | 10 (7) |
| **Which outcomes of DECLINING gastrostomy feeding do you routinely discuss with people with MND?** (N=139) |  |
| Risk of aspiration | 111 (80) |
| Impact on the person's weight | 109 (78) |
| Risk of choking | 106 (76) |
| Impact on quality of life | 104 (75) |
| Time taken to finish meals | 87 (63) |
| Impact on caregivers (e.g. family) | 70 (50) |
| Estimated length of life remaining (prognosis) | 50 (36) |
| I do not discuss outcomes of commencing gastrostomy feeding with people with MND | 8 (6) |
| Other | 10 (7) |
| **What goals of gastrostomy feeding do you typically discuss with people with MND during decision making?** (N=139) |  |
| Weight maintenance/increase | 103 (74) |
| Reduce risk of aspiration | 107 (77) |
| Prevent choking | 89 (64) |
| Provide an alternative route for medication administration | 131 (94) |
| Reduce fatigue | 105 (76) |
| Prolong life | 32 (23) |
| Prevent/slow muscle wasting | 42 (30) |
| Improve quality of life | 107 (77) |
| Reduce caregiver burden | 41 (30) |
| Improve caregivers quality of life | 42 (30) |
| Reduce stress/burden at mealtimes for person with MND | 132 (95) |
| I do not discuss goals of gastrostomy feeding with people with MND | 3 (2) |
| Other | 6 (4) |
| I do not inform people with MND about how long they will have to wait to have the gastrostomy placed | 56 (40) |
| **In your opinion, do healthcare professionals have a responsibility to give recommendations to people with MND about whether or not to have a gastrostomy placed?** (N=139) |  |
| Yes | 72 (52) |
| No | 67 (48) |
| **Do you give recommendations to people with MND about whether or not they should have a gastrostomy placed?**(N=139) |  |
| Never | 42 (30) |
| Rarely | 29 (21) |
| Sometimes | 38 (27) |
| Often | 23 (17) |
| Always | 7 (5) |
| **When you believe the following decisions are best for a person with MND do you give recommendations that they should...** |  |
| **...have a gastrostomy placed?** (N=136) |  |
| Never | 39 (29) |
| Rarely | 24 (18) |
| Sometimes | 42 (31) |
| Often | 21 (15) |
| Always | 10 (7) |
| **...continue without a gastrostomy tube?** (N=137) |  |
| Never | 51 (37) |
| Rarely | 27 (20) |
| Sometimes | 49 (36) |
| Often | 5 (4) |
| Always | 5 (4) |
| **...not ever have a gastrostomy placed?** (N=135) |  |
| Never | 71 (53) |
| Rarely | 30 (22) |
| Sometimes | 28 (21) |
| Often | 3 (2) |
| Always | 3 (2) |
| **Do you discuss the future withdrawal of enteral feeding during decision making discussions about gastrostomy placement?** (N=139) |  |
| Never | 27 (19) |
| Rarely | 26 (19) |
| Sometimes | 43 (31) |
| Often | 27 (19) |
| Always | 16 (12) |
| **Ideally, by what percentage (%) weight loss from symptom onset do you feel people with MND should have had a gastrostomy placed?** (N=124) |  |
|  | Mean 8.1%  SD 3.8  Range: 1-25% |
| **Ideally, in relation to their respiratory function, when do you feel people with MND should have a gastrostomy placed?** (N=138) |  |
| Prior to presenting with any respiratory symptoms or objective signs of respiratory failure | 63 (46) |
| When they have symptoms of respiratory failure but objective respiratory measures remain in normal range | 39 (28) |
| When they have symptoms of respiratory failure and objective respiratory measures are outside normal range | 6 (4) |
| When initiated on non-invasive ventilation (NIV) | 2 (1) |
| When they are using non-invasive ventilation (NIV) for more than 8 hours per 24 hour period | 1 (1) |
| When they are using non-invasive ventilation (NIV) for more than 16 hours per 24 hour period | 0 (0) |
| I do not refer to markers of respiratory failure when making decisions regarding gastrostomy insertion | 27 (20) |
| **Ideally, in relation to their swallowing function, when do you feel people with MND should have a gastrostomy placed?** (N=138) |  |
| Prior to presenting with any swallowing difficulties | 15 (11) |
| When they are noticing swallowing difficulty but not needing to modify the texture of their diet or fluids | 50 (36) |
| When they need to modify the texture of diet or fluids but continue to manage adequate amounts | 61 (44) |
| When they are presenting with adverse consequences of swallowing difficulties e.g. chest infections, choking episodes, reduced intake, prolonged mealtimes | 8 (6) |
| When the consequences of swallowing difficulties present a significant risk to the respiratory health or nutritional intake of the person with MND | 2 (1) |
| I do not refer to markers of swallow function when making decisions | 2 (1) |
| **In your opinion do most people with MND agree to gastrostomy placement:** (N=137) |  |
| Too early | 0 (0) |
| About the right time | 71 (52) |
| Too late | 66 (48) |
| **Do you encounter any challenges communicating with any members of the care team in your local area when supporting people with MND to make decisions about gastrostomy placement?** (N=138) |  |
| Yes | 45 (33) |
| No | 93 (67) |
| **Do you experience these challenges specifically with any of the following professionals?** (N=40) |  |
| Neurologist | 20 (50) |
| Palliative care doctor | 4 (10) |
| Palliative care nurse | 3 (8) |
| Gastroenterologist | 12 (30) |
| Radiologist | 0 (0) |
| Anaesthetist | 1 (3) |
| Respiratory doctor | 10 (25) |
| Respiratory physiotherapist | 3 (8) |
| Respiratory nurse | 2 (5) |
| Physiotherapist (non-respiratory specialist) | 1 (3) |
| MND/neurology Specialist Nurse | 8 (20) |
| MND coordinator | 4 (10) |
| Community neurology matron | 0 (0) |
| Home Enteral Feed Dietitian | 2 (5) |
| Dietitian (non-home enteral feeding) | 2 (5) |
| Speech and language therapist | 4 (10) |
| Nutrition/gastrostomy nurse or advanced clinical practitioner | 2 (5) |
| Occupational therapist | 1 (1) |
| Psychologist | 0 (0) |
| Social worker | 0 (0) |
| General practitioner (GP) | 10 (25) |
| Other | 3 (8) |
| **How effective do you believe the communication is between healthcare professionals in your local area when supporting people with MND to make decisions about gastrostomy placement?** (N=139) |  |
| Not effective at all | 1 (1) |
| Slightly effective | 10 (7) |
| Moderately effective | 40 (29) |
| Very effective | 72 (52) |
| Extremely effective | 16 (12) |
| **In your opinion, how consistent is the information given to people with MND about gastrostomy placement, by different members of your local MND care team?** (N=138) |  |
| Not at all consistent | 5 (4) |
| Slightly consistent | 16 (12) |
| Moderately consistent | 60 (44) |
| Very consistent | 48 (35) |
| Extremely consistent | 9 (7) |

**Supplementary information 6.**

The categories to two questions about participants beliefs about when pwMND should have a gastrostomy placed in relation to the severity of their respiratory failure and dysphagia were combined to allow for the statistical comparisons to be made. The original question and answer categories are displayed in supplementary table 2 below.

**Original data collected.**

| **Ideally, in relation to their respiratory function, when do you feel people with MND should have a gastrostomy placed? (tick one option only)** (N=138) | n (%) |
| --- | --- |
| Prior to presenting with any respiratory symptoms or objective signs of respiratory failure | 63 (46) |
| When they have symptoms of respiratory failure but objective respiratory measures remain in normal range | 39 (28) |
| When they have symptoms of respiratory failure and objective respiratory measures are outside normal range | 6 (4) |
| When initiated on non-invasive ventilation (NIV) | 2 (1) |
| When they are using non-invasive ventilation (NIV) for more than 8 hours per 24 hour period | 1 (1) |
| When they are using non-invasive ventilation (NIV) for more than 16 hours per 24 hour period | 0 (0) |
| I do not refer to markers of respiratory failure when making decisions regarding gastrostomy insertion | 27 (20) |
| **Ideally, in relation to their swallowing function, when do you feel people with MND should have a gastrostomy placed? (tick one option only)** (N=138) |  |
| Prior to presenting with any swallowing difficulties | 15 (11) |
| When they are noticing swallowing difficulty but not needing to modify the texture of their diet or fluids | 50 (36) |
| When they need to modify the texture of diet or fluids but continue to manage adequate amounts | 61 (44) |
| When they are presenting with adverse consequences of swallowing difficulties e.g. chest infections, choking episodes, reduced intake, prolonged mealtimes | 8 (6) |
| When the consequences of swallowing difficulties present a significant risk to the respiratory health or nutritional intake of the person with MND | 2 (1) |
| I do not refer to markers of swallow function when making decisions | 2 (1) |

**The new categories formed to allow the comparative statistics to be undertaken.**

| **Question category from original survey** | **New category** |
| --- | --- |
| **When participants believed gastrostomy tubes should be placed in relation to respiratory function** | |
| Prior to presenting with any respiratory symptoms or objective signs of respiratory failure | Prior to any respiratory symptoms |
| When they have symptoms of respiratory failure but objective respiratory measures remain in normal range  **AND**  When they have symptoms of respiratory failure and objective respiratory measures are outside normal range | Compensating for early respiratory symptoms i.e. pre-NIV |
| When initiated on non-invasive ventilation (NIV)  **AND**  When they are using non-invasive ventilation (NIV) for more than 8 hours per 24 hour period  **AND**  When they are using non-invasive ventilation (NIV) for more than 16 hours per 24 hour period | Experiencing significant consequences i.e. on NIV |
| **When participants believed gastrostomy tubes should be placed in relation to swallowing function** | |
| Prior to presenting with any swallowing difficulties | Prior to any swallowing problems |
| When they are noticing swallowing difficulty but not needing to modify the texture of their diet or fluids  **AND**  When they need to modify the texture of diet or fluids but continue to manage adequate amounts | Compensating for impact of dysphagia e.g. through texture modified diet |
| When they are presenting with adverse consequences of swallowing difficulties e.g. chest infections, choking episodes, reduced intake, prolonged mealtimes  **AND**  When the consequences of swallowing difficulties present a significant risk to the respiratory health or nutritional intake of the person with MND | Experiencing significant consequences e.g. chest infections, choking episodes |

This answers to each question are represented in the main manuscript as table 3 (repeated below), indicating that severity of respiratory failure is a greater driver of participants beliefs about gastrostomy timing than severity of dysphagia. The data in the shaded boxes include those participants who believed that pwMND should have a gastrostomy placed at a similar severity stage i.e. 13 participants believed that gastrostomy should be placed prior to any respiratory symptoms and that gastrostomy should be placed prior to any swallowing problems. The data under the shaded rows includes those participants who believed that pwMND should have gastrostomy tubes placed earlier in relation to respiratory failure than in relation to dysphagia e.g. 48 participants believed that pwMND should have gastrostomy tubes removed prior to any respiratory symptoms but once having to compensate for the impact of dysphagia.

**Table 3 (taken from main manuscript). Participants responses to questions about when they believe pwMND should have a gastrostomy placed in relation to their respiratory and swallowing function.**

|  | **When participants believed gastrostomy tubes should be placed in relation to respiratory function (n (%))** | | | | |
| --- | --- | --- | --- | --- | --- |
| **When participants believed gastrostomy tubes should be placed in relation to swallowing function** | Prior to any respiratory symptoms | Compensating for early respiratory symptoms   i.e. pre-NIV | Experiencing significant consequences   i.e. on NIV | Never refer to respiratory failure | Total |
| Prior to any swallowing problems | 13 (10) | 1 (1) | 0 (0) | 1 (1) | 15 (11) |
| Compensating for impact of dysphagia e.g. through texture modified diet | 48 (35) | 40 (29) | 3 (2) | 20 (15) | 111 (81) |
| Experiencing significant consequences e.g. chest infections, choking episodes | 1 (1) | 4 (3) | 0 (0) | 4 (3) | 9 (7) |
| Never refer to dysphagia | 0 (0) | 0 (0) | 0 (0) | 2 (2) | 2 (2) |
| Total | 62 (45) | 45 (33) | 3 (2) | 27 (20) | 137 |

**Supplementary information 7.**

The topics discussed by the different professions in relation to the placement of a gastrostomy and life on enteral feeding. * = indicates a significant difference (p⩽0.05) between the responses of different professions. The professional role most reporting a topic is indicated in **bold**.

| **Topic discussed with pwMND during decision making…** | **Percentage of HCP discipline stating they discuss the topic (n (%)) N=132** | | | |  |
| --- | --- | --- | --- | --- | --- |
|  | Nurse  (N=23) | Dietitian  (N=73) | Doctor (N=17) | SLT (N=19) | p |
| **…in relation to the placement of a gastrostomy** |  |  |  |  |  |
| The impact of respiratory failure on the procedural risks | 18(78) | 49(67) | **16(94)** | 8(42) | 0.006* |
| Detail about the procedure to place the gastrostomy | 19(83) | **61(84)** | 13(77) | 11(58) | 0.102 |
| The sedation required during the procedure | **18(78)** | 42(58) | 9(53) | 6(32) | 0.025* |
| The length of hospital stay | **22(96)** | 55(75) | 11(65) | 9(47) | 0.004* |
| The risk of aspiration during the procedure | **19(83)** | 41(56) | 6(35) | 13(68) | 0.017* |
| The risk of not surviving the procedure | 12(52) | 28(38) | **10(59)** | 2(11) | 0.012* |
| Pain post gastrostomy placement | **20(87)** | 50(69) | 10(59) | 4(21) | <0.001* |
| Risk of infection post gastrostomy placement | **18(78)** | 50(69) | 7(41) | 11(58) | 0.077 |
| **…in relation to living with a gastrostomy** |  |  |  |  |  |
| Care of the gastrostomy | 21(91) | **66(90)** | 5(29) | 9(47) | <0.001* |
| Care of gastrostomy stoma site | 19(83) | **62(85)** | 4(24) | 3(16) | <0.001* |
| Impact gastrostomy may have on body image | **18(78)** | 33(45) | 5(29) | 3(16) | <0.001* |
| Tube complications and how they are managed | 13(57) | **59(81)** | 3(18) | 5(26) | <0.001* |
| Stoma complications and how they are managed | 13(57) | **59(81)** | 3(18) | 5(26) | <0.001* |
| Gastrointestinal side effects of gastrostomy feeding | 11(48) | **41(56)** | 2(12) | 5(26) | <0.001* |
| The HCP support for pwMND at home | **22(96)** | 68(93) | 9(53) | 14(74) | <0.001* |
| Enteral feeding methods | 22(96) | **71(97)** | 10(59) | 12(63) | <0.001* |
| The impact gastrostomy feeding will have on caregivers | **19(83)** | 54(74) | 4(24) | 9(47) | <0.001* |
| Identifying who will use and care for the tube post placement | 21(91) | **70(96)** | 8(47) | 11(58) | <0.001* |
| The option to withdraw enteral feeding in the future | 14(61) | **48(66)** | 8(47) | 6(32) | 0.043* |

The outcomes discussed by different professions in relation to when discussing **accepting** gastrostomy placement. * = indicates a significant difference (p⩽0.05) between the responses of different professions. The professional role most reporting a topic is indicated in **bold**.

| **Outcome of gastrostomy feeding discussed** | **Discussed in relation to accepting gastrostomy (n (%)) N=132** | | | | **p** |
| --- | --- | --- | --- | --- | --- |
|  | Nurses | Dietitians | Doctors | SLT |  |
| Impact on the person's weight | 18(78) | **65(89)** | 11(65) | 12(63) | 0.021* |
| Risk of aspiration | **17(74)** | 51(70) | 10(59) | 12(63) | 0.717 |
| Risk of choking | **16(70)** | 40(55) | 10(59) | 10(53) | 0.618 |
| Impact on quality of life | **20(87)** | 62(85) | 12(71) | 14(74) | 0.366 |
| Time taken to finish meals | 15(65) | **52(71)** | 11(65) | 13(68) | 0.925 |
| Estimated length of life remaining (prognosis) | 5(22) | 10(14) | **8(47)** | 5(26) | 0.023* |
| Impact on caregivers (e.g. family) | 15(65) | **50(69)** | 8(47) | 10(53) | 0.294 |
| Do not discuss outcomes of accepting gastrostomy | 1(4) | 2(3) | 1(6) | 1(5) | 0.906 |

The outcomes discussed by different professions in relation to when discussing **declining** gastrostomy placement. * = indicates a significant difference (p⩽0.05) between the responses of different professions. The professional role most reporting a topic is indicated in **bold**.

| **Outcome of gastrostomy feeding discussed** | **Discussed in relation to declining gastrostomy (n (%)) N=132** | | | | **p** |
| --- | --- | --- | --- | --- | --- |
|  | Nurses | Dietitians | Doctors | SLT |  |
| Impact on the person's weight | **19(83)** | 58(80) | 11(65) | 14(74) | 0.524 |
| Risk of aspiration | **21(91)** | 55(75) | 12(71) | 17(90) | 0.192 |
| Risk of choking | **20(87)** | 53(73) | 12(71) | 16(84) | 0.395 |
| Impact on quality of life | **21(91)** | 50(69) | 12(71) | 14(74) | 0.191 |
| Time taken to finish meals | 13(57) | **49(67)** | 8(47) | 12(63) | 0.435 |
| Estimated length of life remaining (prognosis) | 11(48) | 17(23) | **12(71)** | 6(32) | 0.001* |
| Impact on caregivers (e.g. family) | **14(61)** | 32(44) | 9(53) | 11(58) | 0.435 |
| Do not discuss outcomes of declining gastrostomy | 2(8.7) | 5(7) | 1(5.9) | 0(0) | 0.622 |
